# Department of Commerce, Bureau of the Census, Washington

**Published:** 1913-12

**Authors:** 


### Department of Commerce, Bureau of the Census, Washington.

November 1, 1913.

*To the Physicians of the South.*

GENTLEMEN: I trust that the following reprint of an editorial in the *Southern Medical Journal* will be of interest to you. The need for accurate vital statistics is especially urgent in the South. This need can be met, at an early date, if the medical profession shall, as a whole, come to appreciate the importance of the registration of births and deaths and hence promote the enactment and aid the enforcement of adequate laws for this purpose.

As a lifelong resident of the South I feel deep personal interest in this movement, and desire most heartily to co-operate with the profession, and with the authorities of the various States, until every Southern State is embraced in the registration area of the United States.

Very truly yours,

WM. J. HARRIS,
Director of the Census.

IMPORTANCE OF VITAL STATISTICS.

It is always difficult to impress people with the importance of doing something from which no beneficial results are immediately visible when the duty assumed or the action performed has been ignored or neglected in the past. Such is the case with physicians when it comes to making regular reports of those items of their business which are included in the general terms, "vital statistics."

The routine work of a busy practitioner crowds out of his mind the necessity for not only recording but reporting to the State health authorities every birth, white or black, every case of infectious disease, and every death from whatsoever cause.

Some reason in this way: "They are born and they die anyhow, and the newspapers tell when we have smallpox, scarlet fever, or cholera, or anything like that, so what is the use of any red tape from me, especially as I get no pay for it?"

Two vital errors exist in such a reflection. First, his report *is* necessary, as well as that of all his professional brethren in his territory, to render the State records authentic. Today our population is in an unsettled frame of mind. A larger proportion of them than ever before are looking southward with longing eyes, dreaming of coming to us to establish homes for themselves and their children away from the dust storms and blizzards of the north and northwest. Thoughtful men, whose citizenship we most desire, do not take such an important step without thorough preliminary investigation. They scan and analyze both State and National reports to learn as thoroughly as possible the condition of our general health, the number and causes of deaths, and the proportion of births of white and colored children. Where these are not available or are imperfect a bad impression is created at the start, so that men of means and of most desirable character when finally ready to personally inspect the different places towards which their investigations have inclined them, pass by the localities wherein the doctors are neglectful of the duty of reporting vital statistics as either suspicious or undesirable. Northern people have an exaggerated conception of the unhealthfulness of the Southern climate, especially as regards malaria and hookworm. Moreover, some have an idea that with us the negroes are always immensely in the majority.

These and many other fallacies would to a great extent be extinguished if all our Southern doctors would make prompt and complete reports to the proper officials as the law requires. It would tend to attract to their respective neighborhoods the most desirable class of immigration, thereby increasing the value of real estate of every description, and naturally expanding their practice. Thus it is readily seen that there *is* a need for every physician to furnish his quota of "red tape," and that it *does* promise remuneration for the slight effort required. This is only one angle from which to view the subject. There are others equally or more important to which the *Journal* intends from time to time to invite the attention of its readers.

The Division of Vital Statistics of the United States Census Bureau, under the direction of Dr. Cressy L. Wilbur, is doing a great work in improving vital statistics. The "model" Census bill should be adopted in every State. It is now pending in several Southern States, and the physicians should use their influence to secure its passage. In a number of Southern States in a year or two after the model Census law has been in operation, those States have had reported ninety per cent of births and deaths, and have therefore been placed in the Registration Area.

It is most important for the future development of the South to have complete and accurate vital statistics.—Reprint from the *Southern Medical Journal,* Mobile, Ala., August, 1913.

